# From urban to alpine: environmental microbial transfer in urban adults – the ALM Study

**DOI:** 10.3389/fpubh.2025.1747693

**Published:** 2026-01-21

**Authors:** Johanna Freidl, Daniela Huber, Michael Bischof, René Zechner, Christina Pichler, Lisa Fickel, Victoria Fischer, Herbert Weingartner, Bela Hausmann, Petra Pjevac, Maximilian Kiefel, Thomas Prinz, Günter Gruber, Arnulf Hartl

**Affiliations:** 1 Institute of Ecomedicine, Paracelsus Medical University Salzburg, Salzburg, Austria; 2 Department Health Sciences, University of Applied Science Salzburg, Puch/Urstein, Austria; 3 Institute of Tourism and Mobility (ITM), Lucerne University of Applied Sciences and Arts (HSLU), Lucerne, Switzerland; 4 Department of Geography and Geology, Paris Lodron University Salzburg, Salzburg, Austria; 5 Joint Microbiome Facility, University of Vienna, Vienna, Austria; 6 Department of Laboratory Medicine, Medical University of Vienna, Vienna, Austria; 7 Centre for Microbiology and Environmental Systems Science, University of Vienna, Vienna, Austria; 8 Environment and Climate Hub, University of Vienna, Vienna, Austria; 9 Lab for Intelligent Data Analytics (IDA Lab), Paris-Lodron University Salzburg, Salzburg, Austria; 10 iSPACE plus GmbH, Salzburg, Austria

**Keywords:** alpine pasture environment, exposome, farm-effect, hematological adaptation, microbiome diversity, nasal microbiome, nature-based therapies, psychological wellbeing

## Abstract

**Background:**

Urbanization is linked to reduced microbial exposure, increased prevalence of lifestyle-related diseases, and diminished psychological resilience. In contrast, traditional alpine farming environments offer high biodiversity and low pollution, potentially promoting restorative health effects. The ALM Study (ALpine Farming and Human Nasal Microbiome Diversity) explored the feasibility and physiological impact of a 7-day immersion in such an environment among previously unexposed (“Alm-naive”) individuals.

**Methods:**

This prospective, single-arm feasibility study was conducted in the Riedingtal Valley, Austria. Twenty-two healthy adults (median age: 30.5 years), with no prior agricultural exposure, participated in a 7-day immersive intervention involving daily alpine farming activities. Biological (nasal swabs, venous blood), physiological (VO₂max), and psychological (WHO-5 psychological wellbeing index, EQ-5D VAS, NR-6) data were collected immediately before and after the intervention. The primary outcome was the change in nasal microbiome diversity (16S rRNA gene amplicon sequencing); secondary outcomes included hematological markers, lipid metabolism, inflammatory parameters, and wellbeing scores. Pre-post changes were analyzed using Wilcoxon signed-rank tests.

**Results:**

Nasal microbiome analysis revealed significant increases in species richness and evenness (*p* < 0.001). In addition, descriptive analyses indicated changes in relative phylum-level composition, with reduced Proteobacteria dominance and variable increases in Firmicutes and Actinobacteriota. Hematocrit (+3.1%, *p* = 0.01), reticulocyte count (+0.39%, *p* < 0.001), and platelet count (+27 G/L, *p* = 0.02) increased significantly, suggesting erythropoietic and immunological activation. Additionally, activation of the immune system became evident, as reflected by a slight but significant rise in CRP (+0.04 mg/dL, *p* = 0.01), in the absence of concurrent changes in IL-6 or leukocyte counts. Total cholesterol (−8.08 mg/dL, *p* = 0.02) and non-HDL cholesterol (−2.00 mg/dL, *p* = 0.01) decreased, VO₂max showed a positive trend (+3.43 mL· kg^−1^·min^−1^, *p* = 0.07). WHO-5 psychological wellbeing scores improved markedly (+12%, *p* < 0.001), while other psychometric scales remained unchanged.

**Conclusion:**

A 1-week immersion in a biodiverse alpine environment was associated with measurable changes in the nasal microbiome, hematological and metabolic profiles, and psychological wellbeing. These findings support both the feasibility and the potential health relevance of short-term, nature-based interventions for urban populations.

## Highlights

- A 7-day stay in a high-biodiversity traditional alpine pasture environment significantly increased nasal microbial richness and evenness, with a compositional shift away from Proteobacteria dominance.
- Participants showed hematological adaptations with increased hematocrit, reticulocytes, and platelets, alongside reduced total and non-HDL cholesterol and immunological activation.
- Subjective wellbeing, assessed via the WHO-5 psychological wellbeing index, improved significantly post-intervention, while other psychometric measures remained stable.
- The ALM Study demonstrated the feasibility of alpine exposure as a nature-based intervention, with high participant adherence, measurable biological responsiveness, and logistical viability for integration into preventive health strategies.

## 1 Introduction

### 1.1 Background and rationale

The accelerating global trend toward urbanization has become a defining determinant of public health in the 21st century (1). By 2018, about 75% of Europe’s population resided in urban environments (2), where exposure to air pollution (3), noise (4), reduced biodiversity (5) as well as psychosocial stress (6) converge to shape a novel epidemiological landscape. Urban living is consistently associated with increased incidence of obesity, type 2 diabetes, metabolic syndrome, asthma, increased cardiovascular risk, systemic inflammation and a broad spectrum of psychiatric conditions (3, 7–9).

In response to this growing burden, the exposome concept has emerged as a transformative framework in environmental and public health sciences. Introduced by Wild (10) and expanded by Vermeulen et al. (11), the exposome encompasses the totality of environmental exposures across the lifespan, including chemical agents, dietary inputs, microbial communities, social networks, and physical activity patterns. Unlike traditional risk models focused narrowly on genetics or isolated exposures, the exposome framework embraces complexity and interconnectivity. It also incorporates the role of microbial deprivation in urban settings as a potentially modifiable risk factor for immune dysregulation and chronic inflammation (12, 13). In addition, the “old friends hypothesis” posits that loss of exposure to diverse environmental microbiota – particularly from natural soils, plants, and animals – undermines immunological tolerance and increases susceptibility to allergic and autoimmune, and psychiatric disorders (14). These insights underscore the need to view microbial deprivation not as a peripheral phenomenon, but as a central element of the modern exposome that modulates immune development and resilience. Consequently, integrating exposome science with One Health and planetary health frameworks marks a necessary paradigm shift (15) – which highlights the need to develop multidimensional prevention strategies that take into account the complexity of real-life exposures and their cumulative health effects.

In this context, nature-based therapies – especially those integrating physical activity in biodiverse settings – could offer promising low-cost strategies for mitigating exposomic disease risk: A growing body of evidence supports the physiological and psychological benefits of “green exercise,” defined as intentional physical activity conducted in natural environments (16). Systematic reviews have shown that green exercise as well as exposure to green spaces – even short-term – improves mood, reduces stress, and enhances parasympathetic activation (17, 18). Furthermore, randomized controlled trials in alpine settings demonstrated improvements in cardiopulmonary fitness, cortisol profiles, and quality of life metrics (19–22), particularly when green exercise was combined with psychosocial interventions (43). The physiological synergy of movement and nature represents an underutilized avenue for public health intervention, particularly in sub-healthy urban populations (23).

Beyond the documented psychophysiological benefits of nature-based physical activity, evidence increasingly points to biodiverse rural settings – particularly traditional farms – as environments that confer additional immunological advantages through complex microbial and ecological exposures (24). Notably, Campbell et al. (25) provided robust epidemiological support for the adult “farm effect,” demonstrating that individuals raised on farms—not merely limited to early childhood – exhibit significantly higher adult FEV₁ and lower prevalence of atopic sensitization, asthma, rhinitis, and bronchial hyperresponsiveness compared to their urban counterparts. Similarly, the results of Song et al. (26) underline the role of early childhood rural environmental exposure in the development of respiratory health. This “farm effect” is largely attributed to early-life exposure to diverse microbial consortia associated with livestock, unprocessed foods, and agricultural environments (27). Whether this protective effect can be partially replicated in adulthood through short-term, immersive exposures to alpine farm environments remains an open hypothesis. Traditional alpine pastures may function as complex health-promoting ecosystems, wherein sustained physical activity, exposure to immunologically relevant environmental microbiota, consumption of minimally processed regional foods, animal-assisted engagement, embodied manual labor, multisensory stimulation, sustained physical activity, high-altitude climatic conditions, and culturally embedded experiences of meaning and coherence converge to promote psychophysiological resilience, immune regulation, and mental well-being- constituting what may be termed the *alpine pasture exposome.*

### 1.2 Objectives

The ALM Study is a single-arm feasibility study investigating the immunological, physiological, and psychological effects of a 7-day nature-based lifestyle intervention in healthy urban adults. Participants were temporarily immersed in traditional alpine pasture environments, characterized by high biodiversity and microbial richness. Framed within the exposome paradigm, the study aims to assess whether short-term exposure to such natural settings can modulate key health-related parameters across biological and psychosocial domains.

This feasibility study is guided by the following specific objectives:

- To quantify shifts in the human associated microbiome, with a particular focus on nasal microbial communities, by comparing samples collected pre- and post-intervention in response to direct environmental microbial exposure associated with alpine terrain, livestock, soil, and vegetation.
- To assess hematologic and immunological responses, including differential blood counts and inflammatory biomarkers such as C-reactive protein (CRP), interleukin-6 (IL-6).
- To assess cardiometabolic reactions, lipid metabolism and physiological assessments – including cardiorespiratory fitness (estimated from the Chester Step Test) – will be evaluated.
- To evaluate subjective well-being and psychological functioning using validated self-report instruments, including the *WHO-5 psychological wellbeing index* and the *EQ-5D-5L* (visual analogue scale), *Satisfaction With Life Scale* and the *Nature Relatedness Scale.*
- To evaluate the feasibility and safety of a 7-day alpine intervention for urban adults without agricultural backgrounds through a participatory stay on traditional alpine mountain pastures.

Through integration of environmental analytics, microbiome profiling, and clinical outcomes, the study aims to establish a mechanistic link between alpine biodiversity and human health. The broader objective is to position the traditional alpine pastures as a translational model for nature-based prevention, embedded within both ecological and cultural landscapes.

## 2 Materials and methods

### 2.1 Trial design

The ALM Study (“Alpine Farming and Human Nasal Microbiome Diversity”) was conducted as a monocentric, prospective, single-arm interventional feasibility study. Given its exploratory nature and limited sample size, the study is explicitly framed as a pilot investigation to inform the design of adequately powered future trials. Participants were enrolled prior to their initial engagement on alpine farms and underwent assessments immediately before and after a 7-day intervention. Designed as an exploratory investigation, the study aimed to examine changes in the nasal microbiome as well as physiological, immunological, and psychological outcomes in response to alpine pasture environmental exposure among previously unexposed (“Alm-naive”) individuals. Ethical approval was granted by the Ethics Committee of the federal state of Salzburg, Austria (1042/2021, 2022-05-02). All participants provided written informed consent prior to study inclusion, in accordance with the Declaration of Helsinki. The study was retrospectively registered in the ISRCTN register.^1^ This article corresponds to the CONSORT extension to pilot and feasibility trials (44) for randomized controlled trials.

### 2.2 Patient and public involvement

No formal patient or public involvement mechanisms were employed in the design or conduct of this study. However, recruitment and dissemination strategies were informed by stakeholder consultations with representatives of the alpine farming sector. While these interactions did not constitute structured participatory research, they supported contextual adaptation of study procedures and ensured alignment with occupational realities in traditional alpine farming.

### 2.3 Changes to trial protocol

Following ethics committee approval, no substantive modifications were made to the study protocol regarding outcomes, inclusion criteria, or core procedures. However, the originally planned 14-day intervention was shortened to 7 days due to logistical constraints. This adjustment was implemented prior to participant enrollment and did not affect the primary outcome measures or the integrity of the overall study design. Minor operational adjustments (e.g., specimen handling logistics, software updates for digital questionnaires) were documented internally and did not influence data quality or consistency.

### 2.4 Trial setting

The ALM Study was conducted in the Riedingtal Valley (Salzburg, Austria, 47.18478, 13.43308), a remote alpine region characterized by traditional small-scale farming and seasonal transhumance practices. The area is known for its ecological integrity, rich biodiversity, and low levels of anthropogenic disturbance, providing an ideal setting to investigate environmental exposures associated with traditional alpine farming (Table 1). The 7-day alpine pasture interventions took place during the alpine farming season in summer 2022. Over the course of the 7-day intervention, participants engaged in daily farming activities alongside local farmers, rotating between various surrounding pastures situated at elevations between 1,500 and 1,700 m.a.s.l. Participants were accommodated at the former pasture “Seppalm” near the valley entrance. All received the same meals during the intervention.

**Table 1:** Alpine pasture profile of the ALM Study.

Section	Characteristic	Low	1 = fully true	2 = rather true	3 = partially	4 = rather true	5 = fully true	High
Structural and environmental features	Size of pasture area	<5 ha (very small)					●	>50 ha (extensive)
	Altitudinal profile	<1,000 m.a.s.l. (low)			●			>2000 m.a.s.l. (alpine high)
	Terrain structure	Steep/difficult access		●				Flat/accessible
	Vegetation density	Sparse (overgrazed, dry)					●	Lush (diverse and vital)
	Landscape openness	Enclosed/forested					●	Open/panoramic
	Biodiversity	Low (monoculture grasses)					●	High (meadow flowers, herbs)
Built and agricultural infrastructure	Building structure	Dilapidated/abandoned					●	Maintained/used daily
	Livestock diversity	None or single species			●			Multispecies (cow, goat, sheep)
	Farming intensity	Mechanized/industrial					●	Traditional/extensive
	Product processing	Absent (milk transported away)				●		On-site (cheese, butter, etc.)
	Presence of humans	Lonely/uninhabited			●			Socially engaged/frequent contact
Sensory and aesthetic quality	Soundscape	Loud (machinery, tourism)					●	Quiet (wind, birds, cattle bells)
	Cleanliness	Garbage/erosion/damage					●	Clean/preserved
	Smell impression	Neutral/unpleasant					●	Aromatic (herbs, hay, animals)
	Color palette	Monotonous (dry or brown)					●	Colorful (flowers, sky, pasture)
	Thermal impression	Cold/windy				●		Warm/sun-exposed
Accessibility and recreation	Trail conditions	Overgrow/steep/difficult					●	Maintained/signposted/flat
	Resting options	No natural seating					●	Natural seats (rocks, stumps, hay)
	Barrier-free access	No (only for hikers)			●			Yes (partially accessible)
	Presence of water	None visible					●	Streams/springs/troughs

### 2.5 Eligibility criteria

Eligible participants were defined as “Alm-naive” adults aged 18 to 65 years who had not previously lived or worked in agricultural environments. Inclusion required the absence of occupational or residential exposure to farming settings within the past 5 years, including formal training in agricultural institutions or cohabitation with individuals engaged in farming. Both male and female participants were eligible. Exclusion criteria included uncontrolled cardiometabolic, pulmonary, psychiatric, or immunological conditions, such as fasting glucose levels ≥ 140 mg/dL, resting systolic blood pressure ≥ 180 mmHg, diastolic ≥ 110 mmHg, or a PHQ-9 score > 9. Additional exclusions comprised active malignancy or recent cancer treatment (within 5 years), use of antibiotics, corticosteroids, or immunosuppressants, as well as a history of drug, nicotine, or alcohol abuse. Recruitment was initiated following ethical approval and conducted primarily via the study website^2^ and social media platforms. Because recruitment was based on an open, voluntary-response approach, the size of the potentially eligible source population and the total number of individuals screened prior to enrolment cannot be validly enumerated. A transparent accounting of the study cohort is therefore provided at the level of assessment for eligibility, enrolment and analysis.

Interested individuals completed a structured online questionnaire covering sociodemographic, occupational, and health-related criteria. Final eligibility was confirmed by clinical investigators during the baseline examination at the Paracelsus Medical University Salzburg. Written informed consent was obtained in person prior to any study procedure; participants received written study information and were given adequate time to ask questions before signing the consent form.

### 2.6 Intervention

**Figure 1:**
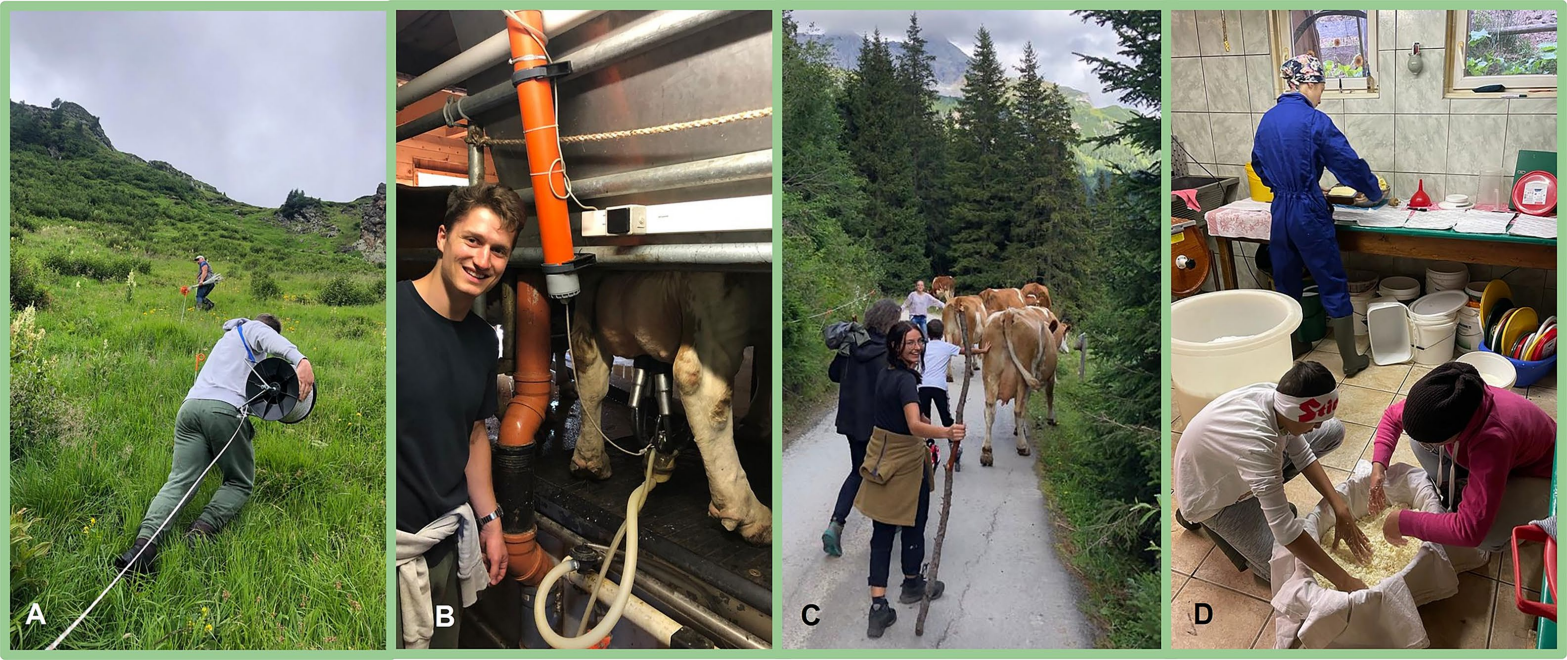
Examples for interventions of the ALM Study. **(A)** Fence in a pasture; **(B)** Milking the cows; **(C)** Driving the cows to the field; **(D)** Processing the milk into butter, curd cheese, and cheese.

The intervention consisted of a 7-day immersive stay in a traditional alpine pasture environment in the Riedingtal Valley (Salzburg, Austria), designed to provide naturalistic exposure to high-biodiversity, low-pollution settings characteristic of Austrian mountain pastures (“Almen”). Participants engaged in daily activities on surrounding pastures, including simple farming tasks performed alongside local farmers. These included milking, cheese production, pasture maintenance, and animal care, offering multimodal environmental exposure without structured work obligations or fixed schedules (examples for interventions see Figure 1). The setting was deliberately non-standardized to preserve ecological validity and simulate real-world conditions. Participants consumed uniform, regionally typical meals throughout the intervention, including unprocessed dairy products, fresh baked goods, and other locally sourced foods, contributing to a culturally and microbially embedded dietary exposure.

The intervention environment was conceptualized as the “Alm-Exposome“, a composite of microbial diversity, natural aerosols, physical activity, solar radiation, and psychosocial seclusion. Data collection occurred exclusively at two time-points: immediately before and after the 7-day intervention. Baseline and post-intervention assessments were conducted either at the Paracelsus Medical University Salzburg (PMU) or directly on-site at the pasture, depending on logistical feasibility. Biomedical parameters (e.g., blood samples), physiological measurements, and psychometric questionnaires were obtained in a standardized manner across both settings. All procedures followed a consistent protocol and were coordinated by the study team at PMU.

### 2.7 Outcomes

All data were collected at two time-points: immediately before (T1) and after (T2) the 7-day intervention. Data were anonymized by four-digit-ID. Biological samples (nasal swabs, venous blood) and psychometric assessments were conducted either at the Institute for Ecomedicine at Paracelsus Medical University Salzburg or directly at the pasture, depending on logistical feasibility.

The primary outcome of the ALM Study was defined as the longitudinal change in human nasal microbiome diversity, assessed via 16S rRNA gene amplicon sequencing.

Alpha diversity was quantified using the Shannon index, richness, evenness and dominance to capture both taxonomic richness and evenness. Observed richness (number of ASVs) was also reported descriptively. Changes between pre- and post-intervention samples were assessed using Wilcoxon signed-rank tests. Microbiome sampling followed validated protocols, and all sequencing analyses were performed anonymously at the Joint Microbiome Facility of the Medical University of Vienna and the University of Vienna.

At each time point, forearm venous blood was collected in Vacuette® tubes (Greiner Bio-One GmbH, Austria) according to the manufacturer’s guidelines. Blood-based secondary outcome parameters included lipid metabolism markers (HDL cholesterol, non-HDL cholesterol, total cholesterol, triglycerides), differential blood count, and inflammatory markers (C-reactive protein and interleukin-6). All laboratory analyses were conducted by the University Institute for Medical and Chemical Laboratory Diagnostics of the Paracelsus Medical University Salzburg (Salzburg, Austria). Cardiorespiratory fitness was assessed using the Chester Step Test (CST), a standardized, externally paced, incremental step protocol in which stepping cadence increases across stages while heart rate and perceived exertion are recorded; these responses were used to estimate VO₂max via validated equations, providing a pragmatic field estimate of aerobic capacity (28). Subjective wellbeing and health-related quality of life were evaluated using validated psychometric instruments: the WHO-5 Psychological Well-Being Index, a 5-item self-report measure of positive wellbeing over the preceding 2 weeks with scores typically transformed to a 0 to100 scale (29); the EQ-5D-5L Visual Analogue Scale (VAS), a single 0 to 100 rating of current overall health anchored from “worst” to “best imaginable health” (30); the Satisfaction With Life Scale (SWLS), a 5-item Likert-rated instrument capturing the cognitive-evaluative component of global life satisfaction (31) and the Nature Relatedness Scale – Short form (NR6), which quantifies enduring affective, cognitive, and experiential connection to nature (32).

### 2.8 Adverse events and harms

No serious adverse events were observed or reported. Minor discomforts related to biological sampling (e.g., nasal swabs, blood draws) were infrequent and self-limited.

### 2.9 Sample size

As a feasibility-focused, exploratory observational study, no formal *a priori* sample size calculation was performed. The primary aim was to assess participant adherence, the variability of key biological and psychosocial parameters in response to naturalistic alpine exposure, and logistical feasibility. Consequently, the planned sample size of 10 to 15 participants was pragmatically determined based on expected recruitment rates, seasonal availability of “Alm-naive” individuals, and logistical constraints associated with repeated *in situ* assessments in remote alpine regions.

### 2.10 Randomization and blinding

No randomization procedures were employed. The ALM feasibility study was conceived as a single-arm, prospective observational trial without group allocation or concealment. Given the nature of the exposure and longitudinal design, neither participants nor investigators were blinded to timepoint or environment. However, laboratory researchers conducting microbiome and cytokine analyses, as well as statistical analysts, were blinded to participant identity through pseudonymized data coding, thus preserving analytical neutrality.

### 2.11 Statistical methods

All statistical analyses and visualizations were performed with R (33), using the libraries mia (34) and ggplot2 (35). Data were assessed for normality using the Shapiro–Wilk test. Given the paired pre-post study design and the small sample size as well as the absence of normality, nonparametric Wilcoxon signed-rank tests were used for all outcome comparisons. Statistical significance was defined as two-tailed *p* < 0.05. Reasons for missing data are described where applicable.

## 3 Results

### 3.1 Participants and baseline characteristics

Recruitment was conducted via advertisements on social media and in local newspapers. A total of 28 individuals were assessed for eligibility, of whom 24 met the inclusion criteria and were allocated to the intervention. During the intervention period, three participants were lost to follow-up—two due to knee problems and one due to sudden hearing loss. In total, 22 participants completed the study. The study cohort comprised 14 (63.6%) female and 8 (36.4%) male participants. At T2, nine participants developed flu-like symptoms and were therefore excluded from the analysis of inflammatory parameters, as acute infections can transiently and strongly alter inflammatory parameters, which would have confounded the interpretation of the intervention effects.

Baseline characteristics are presented in Table 2.

**Table 2:** Baseline characteristics of study participants (*n* = 22).

Variable	Median	Q1	Q3
Age (years)	30.50	24.00	41.00
Height (cm)	173.00	165.00	177.25
Weight (kg)	68.05	58.30	73.73
Systolic BP (mmHg)	118.00	112.75	124.25
Diastolic BP (mmHg)	73.27	68.75	80.50
Heart rate (bpm)	65.50	59.75	69.50
SpO₂ (%)	98.00	97.94	99.00

Values are presented as median and interquartile range. Q1, first quartile (25th percentile); Q3, third quartile (75th percentile). Only participants who completed the study are included in the analysis.

### 3.2 Hematological adaptions, lipid metabolism and inflammatory markers

For hematological and inflammatory markers, a reduced number of participants was included in the analysis, as data from individuals with acute influenza-like illness at day 7 were excluded to avoid confounding effects.

**Figure 2:**
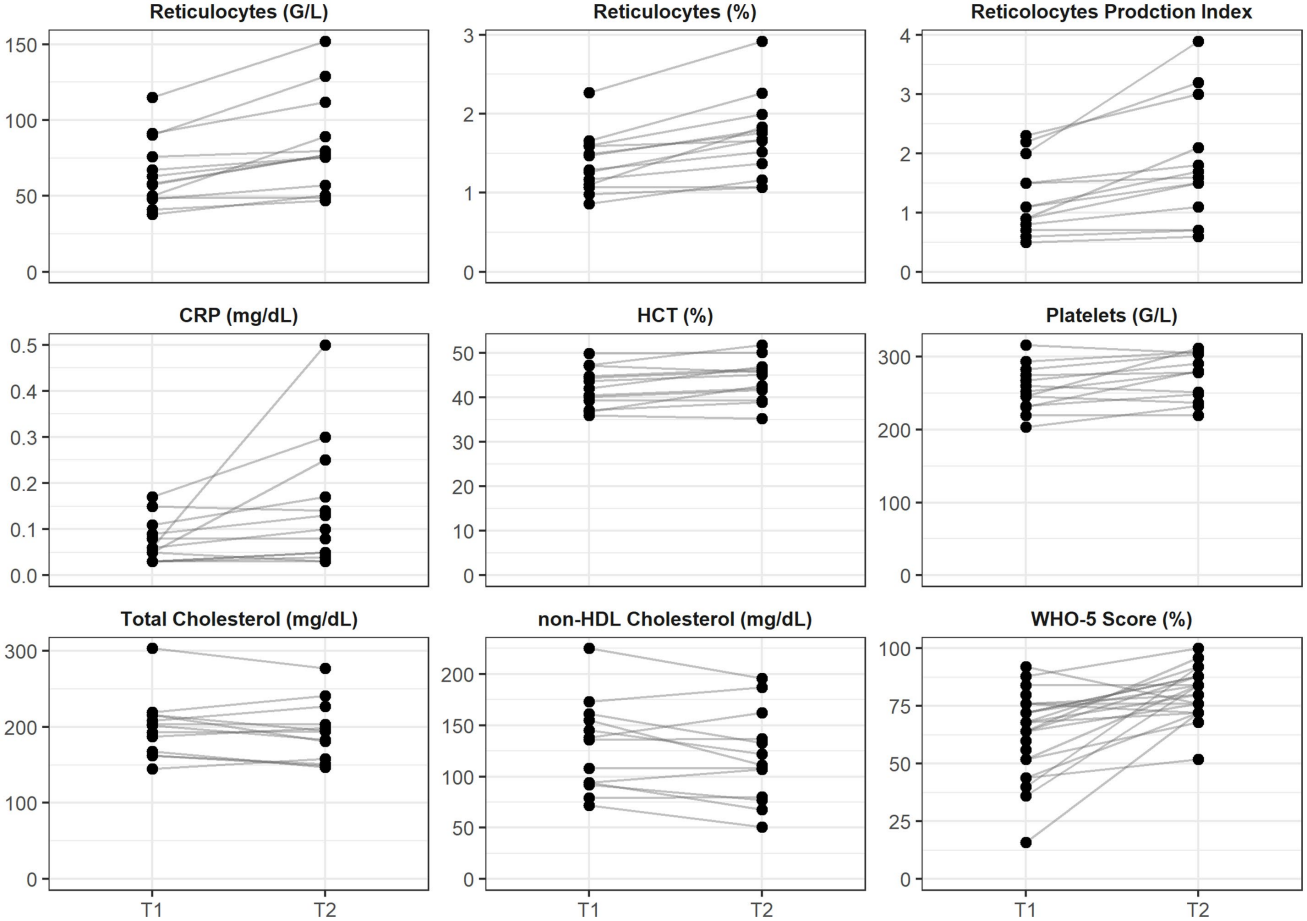
Individual trajectories of hematological, inflammatory, lipid, and psychological parameters before (T1) and after (T2) exposure. Each line represents one participant. Panels are scaled independently with y-axes starting at zero. CRP, C-reactive protein; HDL, high-density lipoprotein; HCT, hematocrit; non-HDL, non–high-density lipoprotein.

Significant alterations were observed in red blood cell indices. The hematocrit rose significantly (Δ + 3.10%, *p* = 0.01, *r* = 0.72), accompanied by a strong increase in reticulocyte count (Δ + 0.39%, *p* < 0.001, *r* = 0.84) and reticulocyte production index (Δ + 0.50, *p* < 0.001, *r* = 0.84). Absolute reticulocytes count also increased (Δ + 18.7 G/L, *p* < 0.001, *r* = 0.84), suggesting an acute stimulation of erythropoiesis. Hemoglobin concentrations exhibited a moderate, non-significant increase (Δ + 0.5 g/dL, *p* = 0.10, *r* = 0.46), while erythrocyte counts remained stable (Table 3; Figure 2).

**Table 3:** Main outcome measures before and after alpine exposure (T1 vs. T2).

Domain	Parameter	*n*	T1 median	Q1	Q3	T2 median	Q1	Q3	Δ median	CI 95%	W	*p*	Effect size (*r*)
Hematological adaptations	Hematocrit (%)	13	42.00	39.30	44.70	45.10	41.60	46.40	3.10	[1, 4.4]	5.50	0.01	0.72
	Reticulocytes (%)	13	1.29	1.10	1.59	1.68	1.37	1.83	0.39	[0.1, 0.6]	0.00	<0.001	0.84
	Reticulocyte Production Index	13	1.10	0.80	1.50	1.60	1.10	2.10	0.50	[0.1, 1]	0.00	<0.001	0.84
	Absolute reticulocyte count (G/L)	13	58.10	48.70	75.80	76.80	57.00	89.10	18.70	[3.9, 29.5]	0.00	<0.001	0.84
	Hemoglobin (g/dL)	13	13.90	13.10	15.50	14.40	13.50	15.40	0.50	[−0.2, 0.8]	14.00	0.10	0.46
	Erythrocytes (T/L)	13	4.60	4.30	5.10	4.60	4.40	5.00	0.00	[−0.1, 0.3]	14.50	0.20	0.36
Lipid metabolism	Total cholesterol (mg/dL)	22	190.08	162.75	216.00	182.00	150.50	208.50	−8.08	[−32, 2.9]	153.50	0.02	0.48
	nHDL (mg/dL)	22	108.50	89.50	146.00	106.50	75.50	134.00	−2.00	[−29.5, 7.5]	173.00	0.01	0.51
	HDL (mg/dL)	22	76.00	64.75	81.21	74.50	64.25	81.25	−1.50	[−6, 5]	88.00	0.79	0.05
	Triglycerides (mg/dL)	22	67.42	54.25	98.25	73.50	57.75	104.25	6.08	[−13, 32.5]	112.50	0.79	0.05
Inflammatory markers	CRP (mg/dL)	13	0.06	0.03	0.09	0.10	0.05	0.17	0.04	[0, 0.1]	5.00	0.01	0.68
	IL-6 (pg/mL)	13	1.50	1.50	2.00	1.50	1.50	1.90	0.00	[−0.5, 0.2]	10.50	0.61	0.14
	Leukocytes (G/L)	13	5.89	5.32	6.36	6.21	5.32	7.25	0.32	[−0.4, 1.3]	27.00	0.37	0.25
	Thrombocytes (G/L)	13	252.00	232.00	274.00	279.00	248.00	303.00	27.00	[−9, 50.9]	9.00	0.02	0.64
Cardiorespiratory fitness	VO2max (mL·kg⁻^1^·min⁻^1^)	22	39.62	33.66	47.68	43.05	34.73	49.40	1.00	[−3.3, 7.9]	50.00	0.07	0.37
Psychological wellbeing	WHO5 (%)	22	68.00	54.00	76.00	80.00	75.00	84.00	12.00	[2, 20]	11.50	<0.001	0.64
	EQ5D VAS (%)	22	80.00	70.00	90.00	90.00	80.00	90.00	10.00	[0, 10]	24.00	0.11	0.33
	FSD (score range 8–56)	22	48.00	43.50	49.50	47.50	45.00	50.25	−0.50	[−2.5, 4]	58.50	0.25	0.24
	SWLS (score)	22	28.00	25.00	30.00	28.00	26.00	31.25	0.00	[−1, 3]	33.00	0.07	0.38
	NR6 (score)	22	4.17	3.75	4.42	4.17	3.96	4.38	0.00	[−0.2, 0.3]	52.00	0.25	0.24

CI, 95% confidence interval; CRP, C-reactive protein; Δ median, change in median from T1 to T2; EQ5D VAS, EQ-5D visual analogue scale; HDL, high-density lipoprotein cholesterol; IL-6, interleukin-6; non-HDL, non–HDL cholesterol; NR6, Nature Relatedness Scale – Short Form; WHO5, WHO-5 psychological wellbeing index; SWLS, Satisfaction With Life Scale; T1, timepoint 1 (pre-intervention); T2, timepoint 2 (post-intervention); VO₂max, maximal oxygen uptake; W, Wilcoxon test statistic; *r*, effect size (Cohen’s *r*).

Among inflammatory markers, C-reactive protein (CRP) increased slightly, but significantly (Δ + 0.04 mg/dL, *p* = 0.01, *r* = 0.68). Conversely, interleukin-6 (IL-6) and leukocyte counts remained unchanged. Notably, platelet count rose significantly (Δ + 27 G/L, *p* = 0.02, *r* = 0.64), which may reflect systemic responses to altitude, exertion, or environmental microbiota exposure. All these parameters can be found in Table 3 and Figure 2.

Total cholesterol decreased significantly by 8.08 mg/dL (*p* = 0.02, *r* = 0.48), and non-HDL cholesterol decreased by 2.00 mg/dL (*p* = 0.01, *r* = 0.51), indicating an amelioration of lipid profile after the intervention. Triglyceride levels exhibited a non-significant upward trend (+6.08 mg/dL), while HDL-cholesterol remained stable (Table 3; Figure 2).

### 3.3 Cardiorespiratory fitness and psychological parameters

Maximal oxygen uptake (VO₂max), a proxy for aerobic fitness, showed a positive trend (Δ + 3.43 mL·kg^−1^·min^−1^), missing statistical significance (*p* = 0.07, *r* = 0.37) (Table 3).

Participants reported a statistically significant improvement in wellbeing as assessed by the WHO-5 psychological wellbeing index (Δ + 12%, *p* < 0.001, *r* = 0.64). EQ-5D-VAS scores also increased (Δ + 10 points), albeit without reaching significance (*p* = 0.11, *r* = 0.33). Other psychological domains such as flourishing (FSD), nature connectedness (NR6), and satisfaction with life scale (SWLS) remained stable (Table 3).

### 3.4 Nasal microbiome

**Figure 3:**
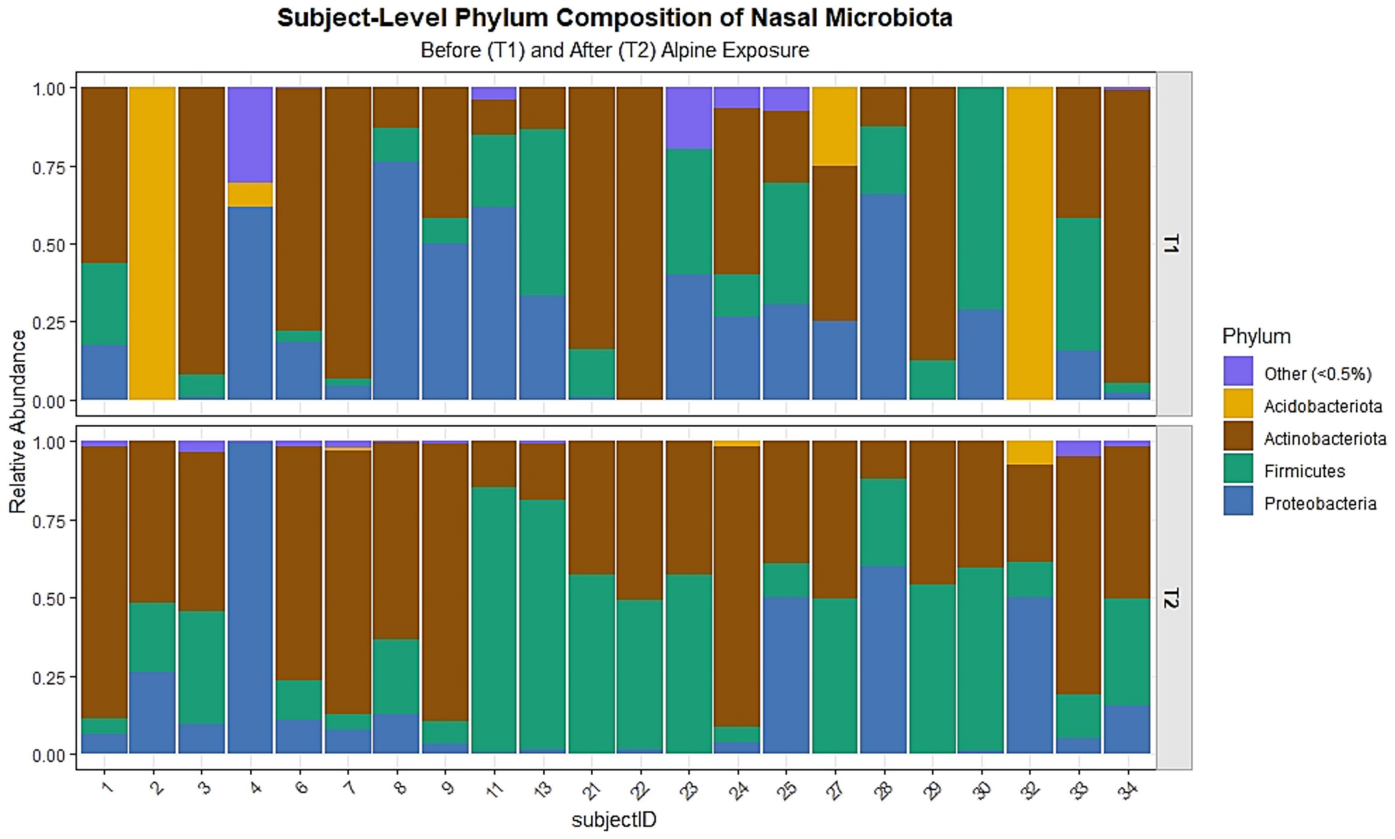
Subject-level phylum composition of nasal microbiota before (T1) and after (T2) alpine exposure. Stacked bar plots depict the relative abundance of bacterial phyla for each participant (subject IDs along the x-axis) at the two sampling time points. Across both time points, the nasal microbiota was dominated by Proteobacteria, Firmicutes, and Actinobacteriota, with smaller contributions from Acidobacteriota, unclassified taxa, and other low-abundance phyla (<0.5%). Following alpine exposure, many participants showed an increased relative abundance of Firmicutes and/or Actinobacteriota, often accompanied by a reduction in Proteobacteria.

Analysis of alpha diversity metrics demonstrated significant post-intervention increases in species richness (observed ASVs, *p* < 0.001) and a significant decrease in evenness (Simpson index, *p* < 0.001), whereas Shannon diversity did not change significantly, consistent with counterbalancing effects of increased richness and reduced evenness (Table 4). Phylum-level analysis of relative abundances (Figure 3) showed that the nasal microbiota at both time points were dominated by Proteobacteria, Firmicutes, and Actinobacteriota, with smaller contributions from Acidobacteriota, unclassified taxa, and other minor phyla (<0.5%). Visual inspection revealed pronounced inter-individual variability at baseline, which largely persisted after the intervention. At the individual level, Figure 3 illustrates heterogeneous paired changes following the intervention. Several participants exhibited increases in the relative abundance of Firmicutes and Actinobacteriota, whereas others showed decreases or no change. In contrast, Proteobacteria, which were abundant at baseline in many participants, tended to decrease post-intervention in a subset of individuals. These patterns are descriptive and reflect visually observed trajectories rather than uniform responses across the cohort.

**Table 4:** Summary of alpha diversity indices for nasal microbiota.

Descriptive statistics						Median difference with CI 95%			Two-sided paired Wilcoxon test		
Parameter (ASVs)	Min	Q1	Median	Q3	Max	T2-T1	CI_low	CI_high	W	*r*	*p*
Richness (observed)	3.00	8.00	11.00	14.00	21.00	5.00	2.50	7.50	176.00	0.75	0.00
Evenness (Simpson)	0.08	0.13	0.22	0.37	0.73	−0.30	−0.45	−0.15	27.00	0.69	0.00
Diversity (Shannon)	0.48	0.75	1.02	1.63	2.09	−0.14	−0.53	0.33	113.00	0.09	0.67
Dominance (relative)	0.23	0.49	0.55	0.70	0.88	0.01	−0.17	0.20	130.00	0.02	0.92

Values are presented as minimum (Min), first quartile (Q1), median, third quartile (Q3), maximum (Max), and interquartile range (IQR). Median differences are reported as T2–T1 with 95% confidence intervals (CI_low, CI_high). *p*-values are from two-sided paired Wilcoxon signed-rank tests comparing T1 and T2. W denotes the Wilcoxon test statistic and *r* the effect size (rank-biserial correlation). The sample comprised 21 participants; ASVs (amplicon sequence variants).

Targeted paired Wilcoxon signed-rank tests were conducted for the four dominant phyla to explore pre–post differences in relative abundance. No statistically significant changes were observed for Acidobacteriota (median Δ = 0.000, V = 22, *p* = 0.205), Actinobacteriota (median Δ = 0.039, V = 97, *p* = 0.346), or Proteobacteria (median Δ = −0.066, V = 167, *p* = 0.194). Firmicutes showed a nominally significant increase (median Δ = 0.124, V = 49, *p* = 0.022); however, this effect did not remain significant after correction for multiple testing (Holm-adjusted *p* = 0.087).

While individual trajectories showed reduced Proteobacteria dominance in several participants, accompanied by increases in Actinobacteriota and Firmicutes in some cases, these changes were heterogeneous and should be interpreted as descriptive trends.

## 4 Discussion

The ALM Study is a feasibility investigation designed to examine immunological, physiological, psychological, and microbiological responses to a 7-day, nature-based lifestyle intervention in healthy urban adults. The alpine trial setting was chosen to capture the full ecological complexity of the “Alm-Exposome,” encompassing diverse environmental, microbial, and climatic factors characteristic of traditional mountain pastures. Conducted in a non-clinical, immersive format, the intervention reflected real-world exposure to a high-biodiversity, low-pollution environment while integrating environmental, physiological, and psychometric assessments.

The intervention elicited clear hematological changes consistent with acute erythropoietic activation. Significant increases in hematocrit, reticulocyte count, reticulocyte production index, and absolute reticulocyte numbers point toward enhanced bone marrow output, likely driven by the combined influence of moderate altitude and sustained physical activity. These changes occurred without pathological alterations and were accompanied by a moderate, non-significant rise in hemoglobin concentration, suggesting a balanced adaptive process rather than excessive erythrocytosis. This systemic adaptation was accompanied by beneficial modulations in lipid metabolism. Total serum cholesterol and non-HDL cholesterol decreased significantly, while HDL cholesterol remained stable and triglyceride levels showed a non-significant upward trend. Although the absolute magnitude of change was modest, the reductions are clinically relevant given the established role of dyslipidemia in atherogenesis (36). These changes likely reflect a composite effect of increased physical activity, dietary simplification, and reduced systemic stress –hallmarks of the traditional alpine agricultural lifestyle.

Inflammatory profiling indicated subtle immune priming without systemic inflammation. CRP increased slightly yet significantly, while IL-6 and leukocyte count remained unchanged. Platelet counts rose significantly, potentially reflecting physiological responses to altitude, sustained exertion, or microbial exposure in a high-biodiversity environment. This selective elevation of acute-phase reactants, without broader inflammatory activation, aligns with the concept of adaptive immune stimulation rather than pathology (37). Such patterns fit within the exposome framework described by Aliberti and Capunzo (38), which posits that environmental exposures, microbiome-mediated immune – metabolic regulation, and psychosocial resilience interact to promote healthy aging and longevity. Physical performance, as measured by VO₂ max via the Chester Step Test, improved by +3,43 mL·kg^−1^·min^−1^ – a borderline-significant change above the threshold of 2.0 mL·kg^−1^·min^−1^ for the minimum clinically important difference (39). The trend suggests early-stage cardiorespiratory adaptation that may be amplified through longer or repeated exposures, offering a potentially scalable strategy to counteract urban-induced deconditioning.

Immersion in the alpine setting significantly improved subjective well-being, as reflected by higher WHO-5 psychological wellbeing index scores. Other psychometric instruments (EQ-5D VAS, Satisfaction With Life Scale, Flourishing Scale) did not show significant change, possibly due to their lower sensitivity to short-term emotional shifts. This pattern is consistent with findings from green exercise and the biophilic exposure hypothesis (45), which suggest that natural environments primarily influence mood and affective states over shorter timeframes.

Nasal microbiota analyses revealed a significant post-intervention increase in species richness, while evenness decreased. Shannon diversity and dominance indices remained unchanged. This pattern indicates that a greater number of taxa became detectable, but their relative abundances were distributed less evenly, without a measurable change in overall diversity. These findings suggest a structural change in community composition; however, they do not allow conclusions regarding microbial stability, resilience, or functional consequences. Taxonomic profiling showed participant-specific changes in dominant phyla, with Firmicutes and Actinobacteriota increasing in some individuals, while others exhibited stable or decreased relative abundances. Although these shifts were not uniform across participants, they are of biological interest in light of previous studies describing Actinobacteriota—particularly *Corynebacterium* spp.—as taxa associated with colonization resistance against respiratory pathobionts such as *Staphylococcus aureus* and *Streptococcus pneumoniae*. Similarly, commensal Firmicutes including *Dolosigranulum* and lactobacilli have been linked to airway immune homeostasis and microbial community balance in earlier work (40). Comparable patterns have been observed in high-biodiversity agricultural settings; for example, dairy farmers have richer nasal microbiota and reduced *Staphylococcus* burden compared with urban dwellers, suggesting competitive suppression of opportunistic colonizers (41). Supporting the broader ecological perspective, Praeg et al. (42) demonstrated that climatic variation, elevation, and soil pH are major microbial diversity drivers in the Italian Alps, while land-use patterns predominantly shape fungal communities. Taken together, these findings indicate that brief immersion in a biodiverse alpine environment is associated with variable changes in nasal microbiota composition, consistent with observations from other biodiversity-rich settings.

The structured survey developed for the ALM Study provided a detailed description of the alpine pasture environment, covering environmental, structural, and sensorial domains. The scoring distribution revealed predominantly favorable scores in biodiversity, pasture maintenance, and traditional infrastructure use (e.g., on-site dairy production, multispecies livestock). Thermal comfort received moderate scores, reflecting the climatic volatility of high-altitude landscapes, while barrier-free access scored low, highlighting infrastructural challenges for inclusive nature-based therapies. The complexity of the exposome – the totality of environmental exposures over the life course – poses a considerable challenge for empirical assessment, particularly in non-clinical, real-world contexts. Microbiome-related health effects are shaped not by isolated variables, but by the interplay of climatic conditions, biodiversity gradients, land-use practices, infrastructure, and sensory stimuli. By systematically capturing these variables, the structured survey offers a reproducible method for integrating environmental parameters with microbiomic, physiological, and psychometric data. This aligns with emerging exposome research frameworks calling for harmonized, multi-domain assessments to contextualize microbiome and health shifts within their broader ecological and physicochemical setting.

### 4.1 Strengths and limitations

A major strength of this study is its integrative, multimodal assessment of physiological, immunological, and psychological responses to a real-world intervention in a high-biodiversity alpine setting. The ecological authenticity of the alpine agricultural environment – marked by microbial diversity, physical activity, and nature immersion – provides a distinctive model of environmental exposure not readily replicated in laboratory conditions.

However, the study is subject to several limitations inherent to its exploratory, single-arm design. The modest sample size, while adequate for feasibility and effect size estimation, limits statistical power and generalizability. Because recruitment relied on voluntary response via online channels, self-selection bias cannot be excluded; participants may have been more health-conscious and/or nature-affiliated than the general population. Additionally, the short duration of the intervention prevents conclusions regarding the sustainability or long-term clinical relevance of observed effects. Moreover, physical activity levels and dietary intake were not standardized, reflecting the real-world conditions of the study setting. The inclusion of participants with influenza-like symptoms in the microbiome analyses represents a potential source of variability. Furthermore, anti-inflammatory immune markers (e.g., IL-10) were not assessed, which limits direct conclusions regarding immunoregulatory pathways. While the inclusion of a control group would be advisable to strengthen causal inference and control for potential confounders, it was deliberately omitted in this initial feasibility phase in order to focus on logistical viability, safety, and biological responsiveness under real-world conditions. Beyond its biomedical relevance, the ALM Study underscores the potential of traditional alpine pasture landscapes as a resource for regional development by linking public health benefits with the valorization of cultural heritage and sustainable land use.

Future work should extend these findings to a larger, adequately powered study – ideally incorporating a control group (e.g., urban control condition or alternative nature exposure), longer exposure durations, and repeated follow-up to characterize persistence of microbiome changes. Multicenter recruitment and systematic environmental measurements (air/settled dust microbiome, particulate matter, and other exposome markers) would strengthen causal inference and external validity.

## 5 Conclusion

In sum, the ALM Study contributes to the growing body of evidence suggesting that structured exposure to biodiverse, low-pollution environments is associated with measurable biological changes across multiple physiological domains within a short timeframe. As a feasibility trial, the study demonstrates the practicability, safety, and participant acceptability of an alpine lifestyle immersion approach, while providing initial signals of biological responsiveness. Although the present findings do not allow conclusions regarding clinical efficacy or long-term health effects, they support the relevance of further investigation in larger, controlled trials. If confirmed, such interventions may represent a complementary, nature-based approach of interest for preventive health research, rehabilitation contexts, and public health–oriented environmental health strategies, particularly in relation to urban-associated physiological and immunological stressors.

## Data Availability

The raw data supporting the conclusions of this article will be made available by the authors, without undue reservation.

## Footnotes

1
https://www.isrctn.com/ISRCTN65561496

2
www.gesunde-alm.com

## Glossary

ASVs: amplicon sequence variants
BP: blood pressure
CRP: C-reactive protein
EQ-5D-5L: EuroQol 5 dimensions
FEV: forced expiratory volume in 1 s
FSD: Flourishing Scale – Short Form
HDL: high-density lipoprotein
HCT: hematocrit
HR: heart rate
IL-6: interleukin-6
non-HDL: non–high-density lipoprotein
NR6: Nature Relatedness Scale – Short Form
PHQ-9: Patient Health Questionnaire-9
RPE: Rating of perceived exertion
SpO₂: Peripheral oxygen saturation
SWLS: Satisfaction With Life Scale
T1/T2: timepoint 1/timepoint 2
VAS: Visual Analogue Scale
VO₂max: maximal oxygen uptake
W: Wilcoxon test statistic
WHO-5 psychological wellbeing index: World Health Organization-Five Well-Being Index
